# Gene expression in a paleopolyploid: a transcriptome resource for the ciliate *Paramecium tetraurelia*

**DOI:** 10.1186/1471-2164-11-547

**Published:** 2010-10-08

**Authors:** Olivier Arnaiz, Jean-François Goût, Mireille Bétermier, Khaled Bouhouche, Jean Cohen, Laurent Duret, Aurélie Kapusta, Eric Meyer, Linda Sperling

**Affiliations:** 1 Centre de Génétique Moléculaire, Université Paris-Sud, CNRS FRE3144, Gif-sur-Yvette, France; 2 Laboratoire de Biométrie et Biologie Evolutive, Université de Lyon, Université Lyon 1, CNRS, UMR 5558, Villeurbanne, France; 3 Institut de Biologie de l'Ecole Normale Supérieure, CNRS UMR8197, INSERM U1024, Paris, France; 4 Department of Biology, Indiana University, Bloomington, IN 47405, USA

## Abstract

**Background:**

The genome of *Paramecium tetraurelia*, a unicellular model that belongs to the ciliate phylum, has been shaped by at least 3 successive whole genome duplications (WGD). These dramatic events, which have also been documented in plants, animals and fungi, are resolved over evolutionary time by the loss of one duplicate for the majority of genes. Thanks to a low rate of large scale genome rearrangement in *Paramecium*, an unprecedented large number of gene duplicates of different ages have been identified, making this organism an outstanding model to investigate the evolutionary consequences of polyploidization. The most recent WGD, with 51% of pre-duplication genes still in 2 copies, provides a snapshot of a phase of rapid gene loss that is not accessible in more ancient polyploids such as yeast.

**Results:**

We designed a custom oligonucleotide microarray platform for *P. tetraurelia *genome-wide expression profiling and used the platform to measure gene expression during 1) the sexual cycle of autogamy, 2) growth of new cilia in response to deciliation and 3) biogenesis of secretory granules after massive exocytosis. Genes that are differentially expressed during these time course experiments have expression patterns consistent with a very low rate of subfunctionalization (partition of ancestral functions between duplicated genes) in particular since the most recent polyploidization event.

**Conclusions:**

A public transcriptome resource is now available for *Paramecium tetraurelia*. The resource has been integrated into the ParameciumDB model organism database, providing searchable access to the data. The microarray platform, freely available through NimbleGen Systems, provides a robust, cost-effective approach for genome-wide expression profiling in *P. tetraurelia*. The expression data support previous studies showing that at short evolutionary times after a whole genome duplication, gene dosage balance constraints and not functional change are the major determinants of gene retention.

## Background

Many diploid animals, plants and fungi are ancient polyploids, with genomes that have undergone one or more rounds of whole genome duplication (WGD). These dramatic events are resolved over evolutionary time by gene loss for the majority of the duplicated genes (reviews: [1,2]). The evolutionary consequences of WGDs have been most studied in model organisms for which functional data are available. In yeast (8% of genes in 2 copies), *Arabidopsis *(33% in 2 copies) and rice (13% in 2 copies), genes that have been retained in two copies usually display functional divergence, reflected in the protein sequence or the expression pattern across different tissues or developmental stages [3-6]. This is consistent with population genetic theory that duplicated genes can be retained on the long term through accumulation of mutations that lead to functional change in one or both copies [7]. The process of retention via partitioning of ancestral functions between the duplicated genes (subfunctionalization) is more likely to occur in species with small effective population sizes, while retention through the fixation of mutations conferring a new function to one of the two copies (neofunctionalization) is more likely to occur in species with high effective population sizes [8], as is typically the case for microorganisms. However, it has been pointed out that duplicate genes fixed through the neutral process of subfunctionalization could subsequently acquire beneficial new functions [9].

Although the immediate consequences of polyploidization can be studied in synthetic polyploid plants created under laboratory conditions [10], it is difficult to investigate events that occur at short evolutionary times after natural WGD. In most ancient polyploids, few genes are still present in 2 copies and those that are have usually undergone neofunctionalization or subfunctionalization. Analysis of early steps in WGD resolution is however possible using *Paramecium tetraurelia*, a unicellular model. This ciliate has nearly 40,000 protein-coding genes as a consequence of 3 successive WGDs [11]. Thanks to a low rate of large-scale genome rearrangement [12], it was possible to identify the paralogs created at each WGD and to establish that 51%, 24% and 8% of the genes duplicated at the recent, intermediate and old WGDs, respectively, are still present in 2 copies in the genome, providing an unprecedented large number of duplicated gene pairs of different ages. (We hereafter refer to the paralogs created by WGD as "ohnologs", to honor the pioneering work of S. Ohno [13] in accord with the proposal of Wolfe [14]).

In *Paramecium*, the initial analysis of the recent WGD [11] indicated that the major determinants of gene retention at relatively short times following a WGD event are gene expression level, as highly expressed genes are retained more than the average, and gene dosage balance, as genes whose products are in complexes are retained more than the average. Efforts to detect subfunctionalization, using the successive WGDs to test the prediction that ohnologs subfunctionalized after a WGD would not be retained in two copies after a subsequent WGD [15], indicated that very little subfunctionalization has occurred in *Paramecium*, and only after long evolutionary times. The whole of the analysis was thus in excellent agreement with the gene balance hypothesis [16,17].

To gain further insight into the evolution of ohnologs after WGD, we have undertaken a genome-wide expression study using custom oligonucleotide microarrays designed for *Paramecium tetraurelia*. The microarray data has already been exploited to show that gene expression is a major determinant of the evolution of gene dosage [18]. The rate of gene loss after WGD turned out to be negatively correlated with gene expression level, not only for the most highly expressed genes (as previously shown using EST data, [11]), but for all levels of gene expression. A new model that takes into account the trade off between the cost and the benefits of gene expression was developed. This COSTEX model predicts that the higher the expression level of the ohnolog pair, the greater the impact of the loss of one copy on fitness, a consequence of the nonlinear function that relates expression level to energetic cost. The model can explain the negative correlation between expression level and rate of gene loss in *Paramecium*, as well as data relating gene dosage to fitness in other organisms such as yeast.

We present here the details of the *Paramecium *microarray platform and gene expression profiling experiments that allowed us to identify genes differentially expressed during 1) the sexual process of autogamy, 2) recovery from deciliation that stimulates ciliogenesis and 3) recovery from massive exocytosis that stimulates biogenesis of new secretory granules known as trichocysts. We examined, for the differentially expressed genes with ohnolog(s), whether functional changes have occurred since the WGD events, as judged by changes in the expression patterns. In agreement with previous analyses, we estimate a very low rate of subfunctionalization, in particular since the recent WGD. A possible evolutionary scenario for resolution of WGD is discussed.

## Methods

### Nimblegen custom microarray design and processing

The design of high density genome-wide microarrays was carried out by NimbleGen (Roche Nimblegen, Madison, WI) using 6 different 50 nt perfect match oligonucleotide probes per conceptual gene transcript, 3 on each strand. Conceptual translation products of the 39,642 gene models annotated during the genome sequencing project [11] were considered to represent all protein-coding gene transcripts, since the only alternative splicing observed in *Paramecium *involves closely spaced alternative splice sites, not exon skipping, and affects only a very small proportion of transcripts [19]. The probes have a mean GC content of 36% although the ORFs in this AT-rich genome have a mean GC content of only 30%. The microarray platform, NimbleGen custom design "2006-09-12_Paramecium", is freely available and has been deposited in GEO [20] (GPL7221, SET01).

NimbleGen's design process optimizes discrimination of related genes. We evaluated the discriminatory power of the microarrays by mapping the probes to the predicted ORFs using BLAT [21]. If we assume that a probe can hybridize to a target sequence with up to 5 mismatches, then we estimate that 91% of the probes hybridize to a unique transcript and 99% to no more than two different transcripts, which are almost always encoded by paralogs that arose from the most recent WGD event. We therefore predict that it will not be possible to discriminate the ~15% of the paralog pairs with the least sequence divergence using the microarray platform.

The microarrays were processed by NimbleGen. RNA samples were reverse transcribed using the Invitrogen Superscript II kit with an oligo-dT primer using a dNTP mix adjusted to take into account the ~70% AT content of *Paramecium *coding sequences. cDNA was labeled with biotin coupled to the Cy3 fluorophore by a terminal transferase reaction. After hybridization and scanning of the microarrays, the probe signals were subjected to RMA background subtraction [22].

### Sample preparation

#### i. Strains and culture conditions

*Paramecium tetraurelia *wild-type reference stocks d4-2 and 51 [23] were used in the exocytosis recovery and autogamy experiments, respectively. Stock 51 carries the wild-type A51 surface antigen gene whereas the largely isogenic stock d4-2 carries the A29 allele. The mutant nd7-1, blocked at a late step of exocytosis owing to a point mutation in the *ND7 *gene [24], was used for reciliation experiments since deciliation triggers exocytosis in the wild type. The mutant nd7-1 is in the d4-2 genetic background.

Cells were grown at 27°C in a wheat grass infusion (BHB, L'arbre de vie, Luçay Le Male, France or WGP, Pines International, Lawrence, KS) bacterised with *Klebsiella pneumoniae *and supplemented with 0.8 μg/ml β-sitosterol according to standard procedures [25].

#### ii. Autogamy

In order to induce the sexual process of autogamy, an autofertilization process during which the germ line micronucleus undergoes meiosis and fertilization to yield a 100% homozygous zygotic nucleus which then gives rise to the new germ line and somatic nuclei (review: [26]), cells were cultured at 27°C under standard conditions. Autogamy was induced by starvation of cultures that had undergone at least 20-25 cell divisions since the previous autogamy. Cell aliquots were removed for total RNA extraction during vegetative growth, after starvation, and over a ~20 hour period during which the new macronucleus develops through programmed rearrangements of the germ line genome in the absence of refeeding. Progression of autogamy was monitored for each time point by staining an aliquot of at least 100 cells with 4',6-diamidino-2-phenylindole (DAPI) followed by fixation in 1% paraformaldehyde - PHEM buffer [27] for observation of nuclear morphology. Six different morphological states were scored for each autogamy time-course sample. "Vegetative" cells have 2 round MICs roughly 2 μm in diameter and a single ovoid MAC roughly 25-30 μm in diameter. The first event during the sexual cycle is "meiosis", scored by observation of MIC division at different stages of meiosis I and II. "Skein" is the next morphological stage, so called because the MAC loses its rounded morphology and appears to unwind, taking on a shape resembling a skein of yarn. The MAC then "fragments" into many small pieces. Note that gene expression continues from the fragments which are only completely lost, by dilution, during the first few vegetative cell divisions after autogamy. The next clearly visible morphological change is the appearance of developing new MACs ("anlagen" stage). Finally, at the end of autogamy, the first cellular division distributes the two new MACs to the daughter cells ("karyonide" stage).

#### iii. Reciliation

In order to induce ciliogenesis, log-phase or stationary cultures of the nd7-1 mutant strain were harvested by centrifugation and deciliation was performed by transfer of 0.2-0.5 ml cell pellets into 7 ml of TrisHCl pH7.4 10 mM, CaCl_2 _1 mM, ethanol 5% in a 15 mL Falcon tube. The cell suspensions were vortexed at maximum speed during 30 sec. The cell suspension was centrifuged 2 min. at low speed in a clinical centrifuge and the cell pellet was returned to fresh culture medium. Cell aliquots were removed for total RNA extraction before (control) and at two times after deciliation.

#### iv. Exocytosis recovery

In order to stimulate massive exocytosis of regulated secretory granules known as trichocysts, cells were cultured under standard conditions at 27°C then harvested by centrifugation. A ~0.5 ml cell pellet was transferred drop by drop to a small beaker containing 5 ml of a solution of 0.05% aminoethyl dextran (AED, [28]) in 10 mM TrisHCl pH 7.2, 1 mM CaCl_2 _under mild rotary agitation to stimulate massive trichocyst discharge. Cells were then rapidly diluted to 100 ml in the same buffer without AED and centrifuged 1 min. at 1000 × g. The pellet, essentially composed of living paramecia having discharged all of their trichocysts were transferred to fresh culture medium. Cell aliquots were removed for total RNA extraction before (control) and at two times after exocytosis.

#### v. RNA extraction

Aliquots of cell cultures containing 2 - 6 × 10^5 ^cells were harvested by centrifugation at 1000 × g. The cell pellet was transferred drop by drop to liquid N_2 _and the frozen cells could be stored at -80°C. Total RNA was extracted from the frozen, unwashed cells using the TRIzol (Invitrogen) procedure, modified by the addition of glass beads during the initial lysis step. After the Trizol/Chloroform treatment, the supernatant was precipitated with isopropanol and the pellet was washed twice in 75% ethanol before final suspension in H_2_O. An aliquot containing 50 - 100 μg of total RNA was precipitated with ethanol and sent in 75% ethanol to NimbleGen for reverse transcription, labeling and hybridization.

### Data analysis

Statistical analysis of the microarray data was carried out using software from the Bioconductor project [29] implemented in the R environment for statistical computing and graphics (R Development Core Team, 2009), version 2.10.0. The limma package version 3.2 was used for differential expression analysis, and the biomaRt package version 2.2, which provides access to the ParameciumDB [30] BioMart advanced query interface [31] from the R environment, for annotation.

#### i. Preprocessing

The probe signals, after RMA background subtraction carried out by NimbleGen or by us using NimbleScan software (version 2.5), were used to evaluate microarray quality by plotting the signal densities. Microarrays with acceptable density profiles (i.e. approximately Gaussian profiles symmetrically centered around a log2 signal intensity value between 9 and 11) were normalized using the quantile method implemented by the normalizeBetweenArrays function of the limma package [32]. The set of microarrays to be used for a given analysis were normalized together (cf. Additional file 1, Table S1). The signals for each gene transcript were determined by taking the median of the 6 corresponding probe signals. The Pearson correlation coefficient r between biological replicate samples, based on the transcript signals, are given in Additional file 1, Table S1, and range from 0.84 to 0.99. The fact that biological replicates had very high correlation coefficients indicates that the technical replication of the experiments was very good. A dot plot comparison of probe signals and gene signals for a pair of biological replicates is shown in Additional file 2, Figure S1.

#### ii. Differential expression

Differential expression was analyzed using the limma package [33]. This involved linear modeling for each gene, use of an empirical Bayes method to moderate the standard errors of the estimated log-fold changes, and correction for multiple sampling using the method of Benjamani and Hochberg [34]. For time-course experiments, we evaluated differential expression across multiple contrasts i.e. we looked for differential expression between any two of the time points in the experiment. Genes were considered to be differentially expressed if the adjusted p-value, equivalent to the false discovery rate (FDR), was less than 0.05.

For analysis of the autogamy data we used TREAT [35], also included in the limma package. TREAT allows introduction into the statistical model of a biologically significant log-fold change threshold, useful for analysis of experiments with a large number of samples since the statistical power can lead to identification of differential expression that is statistically, but perhaps not biologically, significant (i.e. very small log-fold changes). Genes were considered to be differentially expressed for FDR < 0.05 and a model fold-change > 1.5.

#### iii. Hierarchical clustering

Unsupervised hierarchical clustering was carried out using only genes identified as differentially expressed. Pairwise dissimilarities between the samples were calculated as Spearman correlation coefficients and between the genes as Pearson correlation coefficients. The hclust function of R was then used for hierarchical clustering of both samples and genes by the "complete linkage" agglomeration method. The heatmap function was used for graphical representation of the data. The heatmap color scale goes from dark blue for low expression to dark red for high expression.

#### iv. Gene enrichment

In order to evaluate the enrichment of a particular subset of genes, we used the limma package and the eBayes method as described above to evaluate differential expression (p-value < 0.05), except that only the subset of genes under consideration was used to build the expression set, in order to retain maximal statistical power after correction for multiple testing. We tested the null hypothesis of no gene enrichment by randomly selecting 1000 sets of genes of the same size as the subset under consideration, to see how many members of the random subsets were differentially expressed. The enrichment is the ratio of the number of differentially expressed genes in the subset under consideration and the mean number of differentially expressed genes in the 1000 random subsets.

For analysis of Gene Ontology (GO) term enrichment, genes were assigned to GO terms using InterProScan ([36]; August 2008 InterPro database). The Bioconductor globaltest package (version 5.0.1; [37]) was used for testing the association of GO terms with microarray samples, using the default regression model. Multiple testing was corrected according to the method of Benjamani and Hochberg [34].

### Accessing the microarray data

Details concerning the microarray platform used in this study have been deposited at the Gene Expression Omnibus (GEO; [20]) under the accession number GPL7221. SET01 was used for all expression profiling.

The raw probe signals and the signals after RMA background correction have been deposited for each sample under accession numbers GSM315848, GSM315902-GSM315908, GSM365277-GSM365281, GSM447185-GSM447196, GSM450349-GSM450360, GSM450408, GSM450409, GSM450411-GSM450413, GSM450430, GSM450431, GSM450433 and GSM450434. The correspondence between the GEO accession numbers and the samples is given in Additional file 1, Table S1.

The microarray data has also been integrated into ParameciumDB ([30,38]), using the MAGE module of the Chado database schema [39]. The results of the differential expression analyses as well as the raw data can be accessed from each gene page and a track for each microarray experiment showing differential expression can be viewed using the genome browser [40]. It is also possible to use the BioMart advanced query interface [31] to retrieve genes with similar expression profiles (i.e. all genes in a cluster) or to use expression criteria to build up a complex query (for example, find all genes that are up-regulated during meiosis and have an ortholog in human but not in yeast). The reciliation study has also been integrated into Cildb, a knowledgebase about cilia [41,42].

## Results and Discussion

The *Paramecium tetraurelia *microarray platform was designed with NimbleGen Systems to allow expression profiling across the entire genome. Since we wanted to optimize chances of differentiating the expression signals from ohnologs of the recent WGD, 50 mer oligonucleotide probes were chosen. Given the capacity of NimbleGen's high density microarrays at the time when the project started (~400,000 probes per chip) and the large number of *Paramecium *protein-coding genes, 6 probes were designed for each of the 39,642 ORFs predicted by the automated annotation of the genome (see Methods). Although there are errors in this annotation, most often split genes, incorrect translation starts or mistakes in intron identification, the high coding density of the genome (~78%), the small size of *Paramecium *introns (99% are between 20 and 34 nt) and the use of the median of 6 probe signals for each gene signal, all help minimize the repercussions of annotation errors on expression profiling with the platform.

In order to validate the platform and obtain data for analysis of ohnolog expression, we carried out expression profiling of 3 biological processes that have been extensively investigated in *Paramecium*. Each experiment was carried out at least 4 times, by at least 2 different experimenters, in an effort to reduce confounding effects (experimenter, *Paramecium *stock or cell clone, batch of growth medium, etc.). Each experiment and its biological validation is described.

A similar microarray platform was used for genome-wide expression profiling of the life cycle of the only other member of the ciliate phylum with a fully sequenced genome, *Tetrahymena thermophila *[43,44]. Comparison of the *Paramecium *and *Tetrahymena *expression signals for orthologous genes, expression being defined for this purpose as the median across all microarrays, showed good agreement between the two platforms (R^2 ^= 0.33; [18]), especially given the uncertainties in ortholog assignment owing to the fact that *Paramecium *and *Tetrahymena *diverged after the old whole genome duplication, but before the two more recent polyploidization events [11,45].

### i. Autogamy time course identifies developmentally regulated genes

Unique among unicellular organisms, *Paramecium *and other ciliates separate germinal and somatic functions. A germ line micronucleus (MIC) undergoes meiosis and transmits the genetic information across sexual generations. A somatic macronucleus (MAC) contains a rearranged version of the genome streamlined for expression. The genome rearrangements that occur at each sexual generation involve the programmed elimination of 1) ~60,000 short, single-copy germ line DNA elements (IES for Internal Eliminated Sequences) by a precise DNA splicing mechanism and 2) a few hundred regions with germ line repeated sequences, such as transposable elements and minisatellites, by a reproducible but imprecise mechanism that can lead to chromosome fragmentation. DNA elimination is accompanied by uniform endoreplication of the DNA to ~800 haploid copies (reviews: [26,46,47]).

Remarkably, alternative rearrangement patterns in *Paramecium *and other ciliates can be inherited maternally across sexual generations, with no modification of the germ line genome (reviewed in [48]). This is accomplished by a genome-wide subtraction mechanism known as genome scanning. Genome scanning uses short non-coding "scanRNAs", produced from transcripts of the meiotic MIC by a specialized RNA interference pathway [49], to "scan" maternal MAC transcripts [50] through base-pairing. The scanRNAs that do not have cognate sequences in the maternal MAC are transported to the zygotic MAC, where they program DNA elimination, probably through deposit of epigenetic marks on the chromatin.

*Paramecium tetraurelia *can undergo two kinds of sexual processes, conjugation between individuals of opposite mating type, and autogamy, an autofertilization process that yields 100% homozygous progeny. Autogamy, which is induced by starvation of cells that have reached a sufficient clonal age (at least 20-25 vegetative divisions since the last autogamy), can be obtained in mass cultures. However, since cells enter autogamy from a fixed point of the cell cycle, which is not synchronized in the vegetative mass cultures, there is a minimal asynchrony of ~5 hours in this experiment corresponding to the duration of the vegetative growth cycle.

Because of the asynchrony, it was necessary to use nuclear morphology to classify the samples from the independent autogamy experiments for statistical analysis of the microarray signals (Methods). The vegetative time point (VEG) consists of 4 samples from mass cultures containing only log-phase cells showing no sign of meiosis. The meiosis time point (MEI) consists of 4 samples containing 20-39% of cells undergoing meiosis, and little or no fragmentation of the old MAC. The fragmentation (FRAG) time point consists of 4 samples that contained a similar proportion of meiotic cells (20-29%) as the MEI time point, but also contained 37-43% of cells with a fragmented old MAC. The DEV1 time point groups 3 samples with 35-56% of cells with fragmented old MACs and 35-51% of cells that already contained clearly visible new MACs (anlagen). DEV2 consists of 3 samples with 73-98% of cells with visible anlagen, and the DEV3 samples were taken ~10 hours after the DEV2 samples.

**Figure 1:**
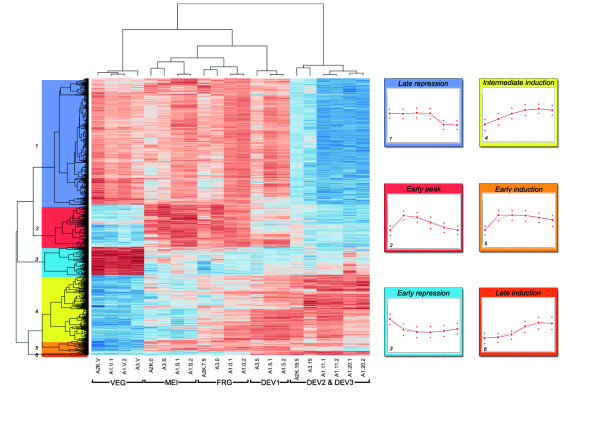
**Hierarchical clusterization of genes differentially expressed during autogamy**. The 2467 genes most differentially expressed during autogamy (treat model fold-change of 2, FDR < 0.05) were hierarchically clustered as described in Methods. The heatmap displays the samples as columns and the genes as rows. The color code goes from dark blue for the lowest expression to dark red for the highest expression. The clusterization of the samples corresponds to the classification based on nuclear morphology, although the DEV2 and DEV3 samples are not resolved. The 6 clusters of co-expressed genes were obtained by cutting the gene dendrogram on the left of the heatmap as indicated. On the right hand side, the average expression profile and standard deviation for each cluster are drawn as they appear in ParameciumDB [65].

Since two of the autogamy time course experiments were carried out in parallel and provide true biological replicates (A1 samples in Additional file 1, Table S1; see also Additional file 2, Figure S1), we used those microarrays to identify a large set of differentially expressed genes independently of any biological classification of the samples. The 12 microarrays of the A1 series, 2 per time point, were normalized together, and for each gene, the minimum and maximum log2 signal intensities across the 12 microarrays were used to extract the 2000 genes with the largest expression difference during autogamy. Then the complete set of autogamy time course experiments, including the A1 biological replicates and the A2 and A3 time series, were normalized together, and the signals from the 2000 selected genes were used for agglomerative hierarchical clustering of the microarrays, using the Spearman correlation coefficient as a proxy for distance. The dendrogram obtained (data not shown, but equivalent to the sample clustering in Figure 1) gave the same sample classification as the nuclear morphology, except for the time points DEV2 and DEV3, which could not be resolved. This could reflect progressive loss of synchrony across the time course or little if any change in gene expression between DEV2 and DEV3, non-exclusive hypotheses. We note that molecular data, not available for all of the microarray samples, can differentiate the DEV2 and DEV3 time points: the relative abundance of double stranded breaks and covalently closed circular intermediates of the IES excision pathway is different [51].

Using the sample classification in 6 time points and treating the microarrays of each time point as biological replicates (cf. Sup Table 1), differential expression was analyzed with different statistical model parameters, as explained in Methods and shown in Table 1. We looked for expression differences between each pair of time points in the series (except for DEV2 and DEV3 which were combined), and identified 5558 genes with biologically (treat model fold-change = 1.5) and statistically (p-value < 0.05) significant differential expression. The results of this analysis have been integrated into ParameciumDB and can be accessed from each gene page. We then chose to hierarchically cluster a somewhat smaller set of 2467 differentially expressed genes (treat model fold-change = 2.0). A heatmap showing the hierarchical clusterization is shown in Figure 1. We used this heatmap to define 6 clusters (Figure 1 and Table 2). Two clusters, "early repression" and "late repression", contain genes that are down-regulated during autogamy. The "late repression" cluster is the largest one and may represent genes that are turned off by complete starvation at the end of the autogamy time course.

**Table 1:** Differential Expression Analysis.

Experiment		Statistical model			Results
Name	Time points	Method	P-Value	Fold-change	Number of genes
Autogamy	6	eBayes	0.05		20164
Autogamy	6	treat	0.05	1.5	5558
Autogamy	6	treat	0.05	2	2467
Reciliation	3	eBayes	0.05		1212
Exocytosis recovery	3	eBayes	0.05		526

The limma package was used for all analyses, with either the eBayes [33] or the treat [35] statistical models and the model parameters (p-Value, Fold-change) indicated. The eBayes model does not incorporate a Fold-change. The p-value is adjusted for multiple testing [34] and is therefore equivalent to a false discovery rate (FDR).

**Table 2:** Clusters of differentially expressed genes.

Experiment	Cluster Name	Number of genes
Autogamy	Early peak	373
	Early induction	97
	Intermediate induction	583
	Late induction	36
	Early repression	252
	Late repression	1126
		
Reciliation	Early peak	264
	Gradual induction	695
	Repression	253
		
Exocytosis	Induced	432
	Repressed	94

The clusters were determined by hierarchical clustering of differentially expressed genes, as shown in Figure 1 for the autogamy experiment. Note that for the autogamy experiment, the 2467 genes identified using a Treat model fold-change of 2 were used for hierarchical clustering.

The four clusters of genes up-regulated at different stages of autogamy were used to validate the experiment. The "early peak" cluster contains genes up-regulated during meiosis while the "early induction" cluster contains genes up-regulated throughout autogamy; "intermediate induction" contains genes induced maximally at the time when the developmental genome rearrangements begin, and "late induction" contains a few genes that are up-regulated only late in development. We compiled a list of developmentally regulated *Paramecium *genes for which published Northern blot measurements of expression during autogamy or conjugation are available. We then looked for these genes in the autogamy clusters. As shown in Table 3, we found good agreement between the published expression data and the autogamy clusters obtained with the microarrays. Spo11 is a conserved endonuclease required for meiosis across all eukaryotes, and the unique *Paramecium *gene is found in the early peak. Other genes expressed early in autogamy are involved in different steps of the genome scanning pathway. Dicer-like genes *DCL2 *and *DCL3 *and the PIWI genes *PTIWI01 *and *PTIWI09 *are involved in the production of the scanRNAs from meiotic MIC transcripts. Nowa1p and Nowa2p are RNA-binding proteins that are thought to be involved in transporting scanRNA from the maternal MAC to the zygotic MAC, and the RNA helicase encoded by *PTMB.220*, ortholog of the *Tetrahymena thermophila EMA1 *gene, could be involved in pairing scanRNA and maternal transcripts as is the case in *Tetrahymena*, where the gene is expressed only during conjugation [52]. The intermediate induction clusters contain the PiggyMac (*PGM*) endonuclease, a domesticated *piggyBac *transposase responsible for the double-stranded breaks that initiate DNA elimination. Other developmentally regulated genes validated by Northern blots have undefined functions, though some have been shown to be necessary for IES excision.

**Table 3:** Developmentally regulated *P. tetraurelia *genes.

Transcript Accession	Synonym	Northern profile	Biological Process	Molecular Function	Cluster Name	Reference
GSPATT00006994001	**NOWA1**	EARLY PEAK	programming DNA elimination	RNA-binding	Early induction	Nowacki et al. 2005
GSPATT00016668001	**NOWA2**	EARLY PEAK	programming DNA elimination	RNA-binding	Early induction	Nowacki et al. 2005
GSPATT00008494001	**DCL2**	EARLY PEAK	programming DNA elimination	ribonuclease III activity	Early peak	Lepere et al. 2008
GSPATT00027456001	**DCL3**	EARLY PEAK	programming DNA elimination	ribonuclease III activity	Early peak	Lepere et al. 2008
GSPATT00021895001	**PTIWI01**	EARLY PEAK	programming DNA elimination	RNA-binding	Early peak	Bouhouche et al. (d)
GSPATT00001395001	**PTIWI03**	EARLY PEAK	?	RNA-binding	Early peak	Bouhouche et al. (d)
GSPATT00020796001	**PTIWI09**	EARLY PEAK	programming DNA elimination	RNA-binding	Early peak	Bouhouche et al. (d)
GSPATT00000299001	**PTMB.220**	EARLY PEAK	MAC development	RNA helicase	Early peak	Nowak et al. 2010
GSPATT00009108001	**SPO11**	EARLY PEAK	meiosis	endonuclease	Early peak	Baudry et al. 2009
GSPATT00007001001	**SUMOI**	EARLY PEAK (c)	DNA elimination	SUMOylation	Early peak	Matsuda et al. 2006
GSPATT00016666001	**SUMOII**	EARLY PEAK (c)	DNA elimination	SUMOylation	Early peak	Matsuda et al. 2006
GSPATT00013187001	**SUMOIII**	EARLY PEAK (c)	DNA elimination	SUMOylation	Early peak	Matsuda et al. 2006
GSPATT00000555001	**PTMB.08**	EARLY PEAK	?	?	not found (a)	Nowak et al. 2010
GSPATT00000151001	**PTMB.344**	EARLY PEAK	DNA mismatch repair	DNA-binding	not found (b)	Nowak et al. 2010
GSPATT00016627001	**PGM**	LATE PEAK	DNA elimination	endonuclease	Intermediate induction	Baudry et al. 2009
GSPATT00024933001	**DIE5a**	LATE PEAK (c)	DNA elimination	?	Intermediate induction	Matsuda et al. 2010
GSPATT00026720001	**DIE5b**	LATE PEAK (c)	DNA elimination	?	Intermediate induction	Matsuda et al. 2010
GSPATT00021288001	**PTIWI08**	LATE PEAK	?	RNA-binding	Intermediate induction	Bouhouche et al. (d)
GSPATT00000552001	**PTMB.10**	GRADUAL INDUCTION	?	?	Intermediate induction	Nowak et al. (e)
GSPATT00000388001	**PTMB.143**	GRADUAL INDUCTION	?	?	Intermediate induction	Nowak et al. (e)
GSPATT00000358001	**PTMB.169**	LATE PEAK	?	?	Intermediate induction	Nowak et al. (e)
GSPATT00000301001	**PTMB.219**	LATE PEAK	?	?	Intermediate induction	Nowak et al. (e)
GSPATT00000022001	**PTMB.443**	LATE PEAK	?	?	Intermediate induction	Nowak et al. (e)
GSPATT00009468001	**PTIWI10**	LATE PEAK	?	RNA-binding	Late induction	Bouhouche et al. (d)
GSPATT00019939001	**PTIWI11**	LATE PEAK	?	RNA-binding	Late induction	Bouhouche et al. (d)

Developmentally regulated genes with published Northern blots are given along with the cluster in which the gene is found in the autogamy experiment, if any. The biological process and the likely molecular function are given when possible. a) This gene figures among the set of 5558 differentially expressed genes, is up-regulated early in autogamy (p-value = 0.003), but was not present in the set of 2467 most differentially expressed genes used for hierarchical clustering. b) This *MSH2 *homolog was not found to be differentially expressed. Examination of the data indicates that the gene is probably up-regulated at meiosis in some, but not all, autogamy experiments. c) The *SUMO *and *DIE5 *Northern blots are of conjugation, not autogamy. d) Bouhouche K, Goût J, Kapusta A, Bétermier M, Meyer E: Functional specialization of Piwi proteins in *Paramecium tetraurelia *from post-transcriptional gene silencing to genome remodeling, submitted; e) Nowak JK, Gromadka R, Juszczuk M, Jerka-Dziadosz M, Maliszewska K, Mucchielli M, Goût JF, Arnaiz O, Agier N, Tang T, Aggerbeck L, Cohen J, Delacroix H, Sperling L, Herbert CJ, Zagulski M, Bétermier M: A chromosome-wide study of genes essential for meiosis and nuclear reorganization in *Paramecium*, submitted. Published data was taken from references [66-69].

### ii. Identification of genes involved in biogenesis of cilia

*Paramecium *has a complex cellular structure. Several thousand cilia anchored at the cell cortex have both motile and sensory functions. They control swimming behavior and feeding activity and mediate reactivity to sexual partners and the environment. The ciliary basal bodies organize the cortical cytoskeleton into a mosaic of cortical units and relay cellular polarities and cell shape through the geometry and timing of their duplication during cell division. Structures equivalent to cilia and ciliary basal bodies were probably present in the last common ancestor of present day eukaryotes, but have been lost in some lineages including most fungi and higher plants [53]. *Paramecium *has long served as a model system for studies of these organelles (review: [47]).

Paramecia can be deciliated without loss of viability and they grow back a full complement of new cilia, as evaluated by electrophysiology of ciliary Ca^2+ ^currents, within about 12 hours [54,55]. RNA samples were prepared before deciliation (control), 30-40 min (early time point) and 120-130 min (late time point) after deciliation. Although the early time point represents maximal transcriptional activation for some of the up-regulated genes, most of the transcripts continued to accumulate at the later time point, so that in addition to clusters of induced and repressed genes, there is a large cluster of "gradually induced" reciliation genes (Table 2).

To validate the set of genes differentially expressed during ciliogenesis, we took advantage of 1108 known ciliary proteins identified by 2 or more peptides in a proteomics study of isolated *Paramecium *cilia [41]. We found that 700 of them (63.2%) are significantly up-regulated in the reciliation experiment (p-value < 0.05). This represents a 25-fold enrichment in known ciliary proteins when compared to a random set of genes (p < 0.001).

### iii. Identification of genes involved in secretory granule biogenesis

*Paramecium*, like exocrine and neuroendocrine cells of vertebrates, has a regulated secretory pathway allowing storage of proteins in dense core vesicles, known as trichocysts, for later release in response to a physiological stimulus. These voluminous storage granules, probably involved in defense against predators, are anchored at the cell cortex at specific docking sites, in a state of pre-membrane fusion (review: [56]). Upon reception of an appropriate external stimulus, synchronous exocytosis of the ~2000 trichocysts can occur within milliseconds. A complete new complement of secretory granules is then synthesized, transported to the cell cortex and docked in an exocytosis-competent state within 6 to 8 hours [57]. RNA samples were prepared before, 40 minutes and 210 minutes after massive exocytosis, allowing identification of clusters of induced and repressed genes (Table 2).

To validate this experiment, we took advantage of a large multigene family that encodes trichocyst cargo proteins. The TMPs (Trichocyst Matrix Proteins) have been well characterized [58,59], and it was shown by nuclear run-on experiments that TMP transcription is induced by exocytosis [60]. We therefore analyzed enrichment of the 176 annotated TMP genes in the exocytosis recovery experiment and found 118 (67%) of the TMPs to be up-regulated after exocytosis, representing a 60-fold enrichment compared to a random set of genes (p < 0.001).

The TMPs do not account for the whole set of genes up-regulated after exocytosis. We also found many genes involved in early steps of the secretory pathway as could be anticipated given our knowledge of trichocyst biogenesis (review: [56]). We therefore undertook GO term enrichment analysis. We found that 7 of the 9 Biological Process terms with the most statistically significant enrichment (p < 0.001) were highly pertinent to trichocyst biogenesis: "ER to Golgi vesicle-mediated transport" (23 genes are associated with this GO term), "regulation of pH" (23 genes), "protein secretion" (9 genes), "protein targeting" (8 genes), "transport" (617 genes), "vesicle-mediated transport" (179 genes) and "SRP-dependent cotranslational protein targeting to membrane" (27 genes). The two other enriched terms, for biosynthetic processes, involved few covariates (1 gene and 7 genes respectively for "lysine biosynthetic process via diaminopimelate" and "polyamine biosynthetic process").

### iv. Independence of the biological processes

**Figure 2:**
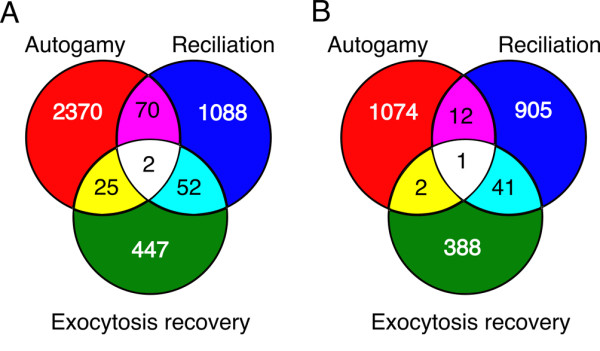
**Genes differentially expressed in more than one experiment**. The Venn diagrams show the overlap between differentially expressed genes identified by each of the 3 experiments. For the autogamy experiment, the set of 2467 most differentially expressed genes was used. A) All differentially expressed genes B) Up-regulated genes.

We examined the overlap between the expression profiles of the 3 experiments, to see whether we could detect genes that are differentially expressed in more than one experiment, such as stress-induced genes. Figure 2 shows the number of differentially expressed genes common to any 2 or all 3 of the experiments (Figure 2A) and the induced genes common to any 2 or all 3 experiments (Figure 2B). The small number of overlapping genes shows that we are not simply detecting stress-induced genes and underscores the independence of the three biological processes, thus providing further validation of the microarray resource.

We examined available annotations for the 42 genes that are up-regulated during both reciliation and exocytosis recovery. None of the structural proteins specific to trichocysts or cilia and none of the membrane trafficking proteins up-regulated during exocytosis recovery were found in the overlapping set. Although half of the 42 genes have unknown functions, many of the remaining ones encode excellent candidates for involvement in shared transcription regulation networks. Six kinases including 2 Ca^2+^-dependent kinases and a cAMP-dependent kinase, a phosphatase, a Ca^2+^-dependent sarco/endoplasmic reticulum ATPase and a phosphatidylinositol-4-phosphate-5-kinase could be involved in transmission of signals from the cell cortex to the nucleus. Four putative transcription factors and a poly ADP-ribose polymerase could be nuclear targets of the signal transduction.

### v. Evolutionary fate of ohnologs of differentially expressed genes

One of the questions that motivated this microarray study was whether or not ohnologs, in particular of the recent WGD, would have different expression patterns, indicative of functional change. We first evaluated the rate of retention of ohnologs of differentially expressed genes for each of the WGD events (Table 4). The retention rates of genes differentially expressed in each biological process are compared to the genome wide values for all 39,642 protein coding genes. The differentially expressed genes appear to have a significantly higher retention rate after the recent WGD than the genome-wide average. Previous analyses showed that the two major determinants of gene retention after WGD are expression level, which is positively correlated with retention, and stoichiometric constraints for genes whose products belong to protein complexes [11,18]. We found that the retention rates for the differentially expressed genes can be, at least partially, explained by expression level, consistent with the COSTEX model [18]. More specifically, we determined that the clusters with a higher retention rate than the genome-wide average, also had a higher basal expression level than the average, as defined fore each gene by the median expression across all microarrays corresponding to control samples (data not shown). The higher than average retention rate for genes differentially expressed during reciliation and exocytosis recovery is in addition consistent with the many genes in the clusters that encode structural components of axonemes or secretory granule contents that assemble together during biogenesis of these edifices.

**Table 4:** Retention of differentially expressed genes after whole genome duplication.

	WGD1	WGD2	WGD3	Genes
Differentially expressed genes				
Reciliation	67%	28%	7%	1212
Exocytosis recovery	68%	32%	9%	526
Autogamy	69%	35%	9%	2467
Genome	61%	32%	8%	39642

The percentage of genes differentially expressed during the biological processes studied that have retained at least one ohnolog from a whole genome duplication event is given along with the total number of genes in the category ("Genes"). The last row in the table shows across the whole genome the percentage of genes that have retained ohnologs after each of the WGD events. WGD1, recent whole genome duplication; WGD2, intermediate whole genome duplication; WGD3, old whole genome duplication. Note that a given gene can have 0 or 1 ohnolog from the recent WGD, 0 to 2 ohnologs from the intermediate WGD and 0 to 4 ohnologs from the old WGD.

We next asked whether the ohnologs of differentially expressed genes would be found in the same cluster, as expected for genes that have the same function. The results (Table 5) are presented not only for the recent WGD but also for the more ancient events. If we consider only the recent WGD (designated WGD1 in the table), then the striking result is that very few ohnologs (between 5.1 and 7.6% depending on the experiment) are found in different clusters, the majority being either in the same cluster or in no cluster at all. The proportion of differentially expressed genes with an ohnolog in a different cluster increases to 8.2 - 12.4% for the intermediate WGD and to 15.3 - 16.6% for the ancient WGD. When examined case by case, not all of the ohnologs in different clusters have significantly different expression patterns indicative of subfunctionalization. For example, ohnologs in "early peak" and "early induction" autogamy clusters are scored as different, but the difference in expression pattern is too small for such cases to considered as subfunctionalization. The values in Table 5 must therefore be taken as upper envelopes. Nonetheless, we could identify a few examples of recent sub-functionalization. Additional file 3, Figure S2 shows the expression patterns during autogamy of ohnologs encoding putative membrane-anchored leucine-rich repeat proteins. One ohnolog is in the early peak cluster, the other in the intermediate induction cluster. The case for subfunctionalization is supported in this example by an outgroup i.e. ohnologs from the intermediate WGD that are not differentially expressed during autogamy.

**Table 5:** Differential expression of ohnologs.

Category	AUTOGAMY	RECILIATION	EXOCYTOSIS
Genes with WGD1 ohnolog	1695	817	355
*same cluster*	*956 (56.4%)*	*248 (30.3%)*	*116 (33%)*
*different cluster*	*92 (5.4%)*	*62 (7.6%)*	*18 (5.1%)*
*not in cluster*	*647 (38%)*	*507 (62%)*	*221 (62%)*
Genes with WGD2 ohnolog	875	339	170
*both in same cluster*	*178 (20%)*	*11 (3.2%)*	*19 (11%)*
*one in same cluster*	*256 (29%)*	*52 (15.3%)*	*30 (17.6%)*
*different cluster*	*94 (10.7%)*	*42 (12.4%)*	*14 (8.2%)*
*none in cluster*	*355 (40.1%)*	*238 (70%)*	*107 (63%)*
Genes with WGD3 ohnolog	205	85	44
*one or more same cluster*	*76 (37%)*	*3 (3.5%)*	*2 (5%)*
*different cluster*	*34 (16.6%)*	*13 (15.3%)*	*7 (15.9%)*
*none in cluster*	*101 (49.2%)*	*69 (81%)*	*36 (82%)*

The ohnologs of differentially expressed genes were examined to see whether they belong to the same cluster, a different cluster, or were not identified as differentially expressed, for each of the three biological processes studied. For WGD3, genes were scored as "same cluster" or as "different cluster" if at least one ohnolog was found in the same cluster or in a different cluster, respectively. WGD1, recent whole genome duplication; WGD2, intermediate whole genome duplication; WGD3, old whole genome duplication.

A substantial proportion of the ohnologs of genes differentially expressed in each experiment is not found in any cluster. Closer examination of some of the cases indicated that this can often be explained by the fact that the ohnolog is actually in an early stage of pseudogenization through gradual decay of its coding sequence, as evaluated by sequence alignment. This is in agreement with the estimate that at least 1500 recent pseudogenes are present in the genome annotations [11]. Additional explanations of why ohnologs of differentially expressed genes are not found in a cluster are limited statistical power so that differential expression was only detected for one ohnolog even though both are (or both are not) differentially expressed, and in a few cases, annotation errors. For the autogamy experiment, since only the 2467 most differentially expressed genes were used for hierarchical clustering (cf. Table 1), some ohnologs that are not in a cluster nonetheless have a similar pattern of differential expression.

Studies of gene duplicates in yeast first showed that regulatory sequences evolve independently of coding sequences in duplicated genes [61] and suggested that changes in expression level are the first step in gene retention [62]. However a majority of the duplicates studied were not the result of polyploidization, but of other, smaller-scale duplication events. In *Paramecium*, it has been shown that gene expression level is a major (but not the unique) determinant of gene retention after polyploidization [18], using the same *P. tetraurelia *microarray resource described here. That study demonstrated that gene expression is strongly correlated with retention of duplicates across the whole range of expression levels measured by the microarrays, and the relationship was strongest with respect to retention after the recent WGD.

We suggest that a likely scenario for the resolution of WGD would begin, at relatively short times after polyploidization, with the fixation of mutations in regulatory sequences. Indeed, analysis of synonymous (Ks) and non-synonymous (Ka) codon substitution rates showed that the coding sequence of ohnologs of the recent *P. tetraurelia *WGD is subject to strong negative selection (Ka/Ks << 1; [11]). However small changes in expression level might be tolerated. Eventually, dosage balance constraints could be relieved by differences in expression level of duplicate genes. As a consequence, the less expressed duplicate would become free to fix mutations in the coding sequence. Most of the resulting functional changes would correspond to a loss of function leading to gene loss through gradual decay of the coding sequence no longer subject to any selective constraints. We expect that future work using technologies that allow direct measurement of the expression level of each ohnolog in a sample, such as RNA-seq, along with studies of regulatory sequences, will make it possible to more rigorously test the postulated scenario for resolution of WGD.

## Conclusions

We have designed a microarray platform for *Paramecium tetraurelia *and used it to generate genome-wide expression data for the first time in this ciliate. Expression profiles of 1) the sexual process of autogamy, 2) reciliation and 3) exocytosis recovery have been integrated into ParameciumDB and all of the microarray data has been deposited in GEO, thus constituting a public microarray resource. Biological validation of the microarray resource was obtained using Northern blots of developmentally regulated genes (Table 3 and references therein), proteomics data for cilia [41] and morphological, immunocytochemical and molecular data concerning secretory granule biogenesis and secretory protein expression [58-60,63]. The microarray resource has already been used to study retention of metabolic genes after WGD [64] and to show that expression is a major determinant of the evolution of gene dosage [18]. Use of the clusters of differentially expressed genes identified here to evaluate functional changes that have occurred since WGD confirms a very low rate of subfunctionalization, especially at short evolutionary times. Although recently developed deep sequencing approaches for the analysis of cellular RNA provide a wealth of information not accessible using microarrays, we consider that the platform described here remains a robust and cost-effective approach for most genome-wide expression profiling applications in *P. tetraurelia*.

## Authors' contributions

Conceived and designed the experiments: JC, MB, EM, LD; executed the experiments: JC, KB, MB, AK; analyzed the data: JFG, OA, EM, LD, LS; deposited the data in GEO: JFG; integrated the data in PararmeciumDB: OA; wrote the paper: LS, OA, JFG. All authors read and approved the final manuscript.

## Supplementary Material

Additional file 1 **Table S1. Microarrays**. This table provides the correspondence between GEO accession numbers, GEO microarray labels and information about the strain, the experiment and the correlation coefficients found for the expression signals of biological replicate microarrays. Click here for file

Additional file 2 **Figure S1. Microarray biological replicates**. Dot plots of log-transformed probe expression signals (left) and transcript expression signals (right) for a pair of biological replicate microarrays. Each transcript signal is the median of the 6 corresponding probe signals. Click here for file

Additional file 3 **Figure S2. Subfunctionalization of ohnologs of the recent WGD**. Log-transformed autogamy time course for a family of 4 ohnologs, taken from ParameciumDB gene pages. The colored bars represent different biological replicates for each time point (see ParameciumDB gene pages for details). From top to bottom, the ParameciumDB accession numbers are GSPATG00035959001, GSPATG00008040001, GSPATG00007828001 and GSPATG00005693001. Only the top two genes are differentially expressed during autogamy, and are found in the "early peak" and "intermediate induction" clusters respectively. The dendrogram drawn on the left indicates the recent and intermediate WGD relationships of the 4 genes. Click here for file
