# Supplementary material for: Gene expression in a paleopolyploid: a transcriptome resource for the ciliate Paramecium tetraurelia
Source: BMC Genomics. 2010 Oct 8;11:547. doi: 10.1186/1471-2164-11-547 (PMC3091696; doi:10.1186/1471-2164-11-547)
Supplement: Additional file 1 — Table S1. Microarrays. This table provides the correspondence between GEO accession numbers, GEO microarray labels and information about the strain, the experiment and the correlation coefficients found for the expression signals of biological replicate microarrays. [file 1471-2164-11-547-S1.PDF]

Accession	Label	Strain	Experiment	Time points	Correlation r
GSM315848	Recil.lp.T0.1	nd7 F2 1A5a	Ciliogenesis	control	0.93 – 0.97
GSM315902	Recil.lp.T0.2	nd7 F2 1A5a	Ciliogenesis	control	
GSM315903	Recil.lp.T0.3	nd7 F2 1A5a	Ciliogenesis	control	
GSM315904	Recil.lp.T0.4	nd7 F2 1A5a	Ciliogenesis	control	
GSM365277	Recil.st.T0.1	nd7 F2 1A5a	Ciliogenesis	control	
GSM315907	Recil.lp.T30.1	nd7 F2 1A5a	Ciliogenesis	early	0.84 – 0.95
GSM315908	Recil.lp.T30.2	nd7 F2 1A5a	Ciliogenesis	early	
GSM365280	Recil.st.T45.1	nd7 F2 1A5a	Ciliogenesis	early	
GSM365281	Recil.st.T45.2	nd7 F2 1A5a	Ciliogenesis	early	
GSM315905	Recil.lp.T120.1	nd7 F2 1A5a	Ciliogenesis	late	0.86 – 0.94
GSM315906	Recil.lp.T120.2	nd7 F2 1A5a	Ciliogenesis	late	
GSM365278	Recil.st.T130.1	nd7 F2 1A5a	Ciliogenesis	late	
GSM365279	Recil.st.T130.2	nd7 F2 1A5a	Ciliogenesis	late	
GSM447185	Trich.T0.1	7s d4-2 self VB mt7-2A	Exocytosis	control	0.88 – 0.94
GSM447191	Trich.T0.3	7s d4-2 self VB mt7-2A	Exocytosis	control	
GSM447194	Trich.T0.4	7s d4-2 self VB mt7-2A	Exocytosis	control	
GSM447186	Trich.T40.1	7s d4-2 self VB mt7-2A	Exocytosis	early	0.90 – 0.95
GSM447189	Trich.T40.2	7s d4-2 self VB mt7-2A	Exocytosis	early	
GSM447192	Trich.T40.3	7s d4-2 self VB mt7-2A	Exocytosis	early	
GSM447195	Trich.T40.4	7s d4-2 self VB mt7-2A	Exocytosis	early	
GSM447187	Trich.T210.1	7s d4-2 self VB mt7-2A	Exocytosis	late	0.89 – 0.97
GSM447190	Trich.T210.2	7s d4-2 self VB mt7-2A	Exocytosis	late	
GSM447193	Trich.T210.3	7s d4-2 self VB mt7-2A	Exocytosis	late	
GSM447196	Trich.T210.4	7s d4-2 self VB mt7-2A	Exocytosis	late	
GSM450349	A1.V.1	51 new	Autogamy	vegetative	0.95 – 0.98
GSM450355	A1.V.2	51 new	Autogamy	vegetative	
GSM450430	A2K.V	51 new	Autogamy	vegetative	
GSM450408	A3.V	51 new	Autogamy	vegetative	
GSM450350	A1.S.1	51 new	Autogamy	meiosis	0.88 – 0.99
GSM450356	A1.S.2	51 new	Autogamy	meiosis	
GSM450431	A2K.0	51 new	Autogamy	meiosis	
GSM450409	A3.S	51 new	Autogamy	meiosis	
GSM450351	A1.0.1	51 new	Autogamy	fragmentation	0.87 – 0.99
GSM450357	A1.0.2	51 new	Autogamy	fragmentation	
GSM450433	A2K.7.5	51 new	Autogamy	fragmentation	
GSM450411	A3.0	51 new	Autogamy	fragmentation	
GSM450352	A1.5.1	51 new	Autogamy	dev1	0.93 – 0.99
GSM450358	A1.5.2	51 new	Autogamy	dev1	
GSM450412	A3.5	51 new	Autogamy	dev1	
GSM450353	A1.11.1	51 new	Autogamy	dev2	0.97 – 0.99
GSM450359	A1.11.2	51 new	Autogamy	dev2	
GSM450434	A2K.19.5	51 new	Autogamy	dev2	
GSM450354	A1.20.1	51 new	Autogamy	dev3	0.91 – 0.98
GSM450360	A1.20.2	51 new	Autogamy	dev3	
GSM450413	A3.15	51 new	Autogamy	dev3	

Table S1. Microarrays
