# Supplementary figures and images for: Gene expression in a paleopolyploid: a transcriptome resource for the ciliate Paramecium tetraurelia
Source: BMC Genomics. 2010 Oct 8;11:547. doi: 10.1186/1471-2164-11-547 (PMC3091696; doi:10.1186/1471-2164-11-547)

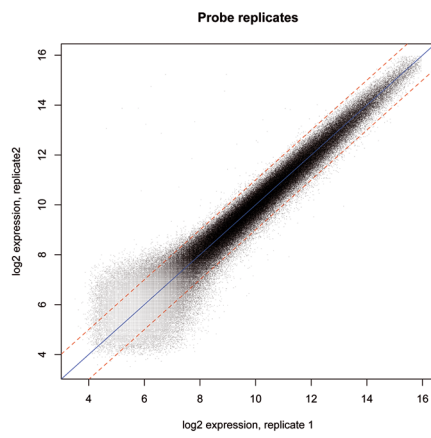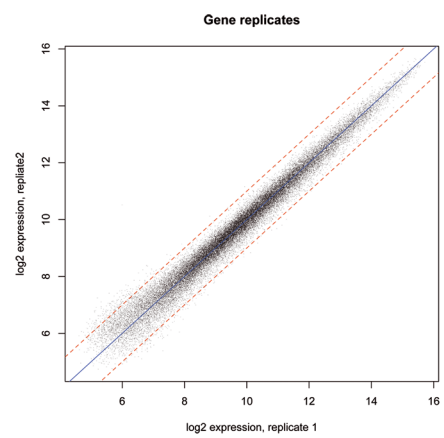

Supplement: Additional file 2 — Figure S1. Microarray biological replicates. Dot plots of log-transformed probe expression signals (left) and transcript expression signals (right) for a pair of biological replicate microarrays. Each transcript signal is the median of the 6 corresponding probe signals. [file 1471-2164-11-547-S2.PDF]

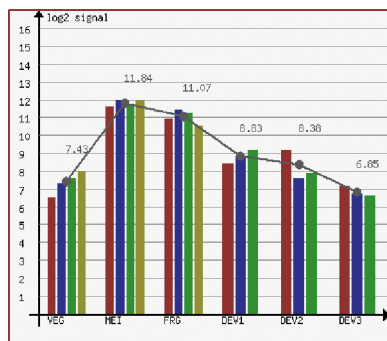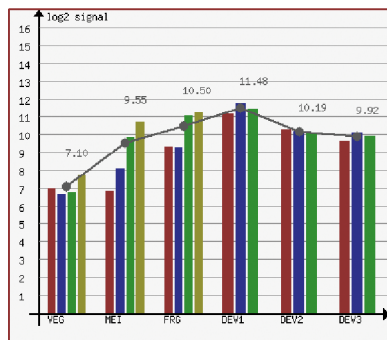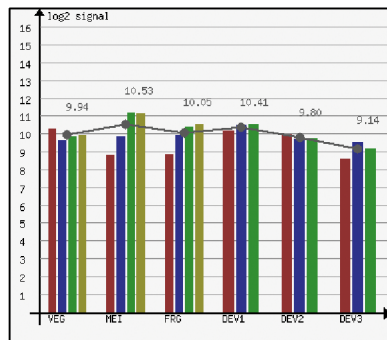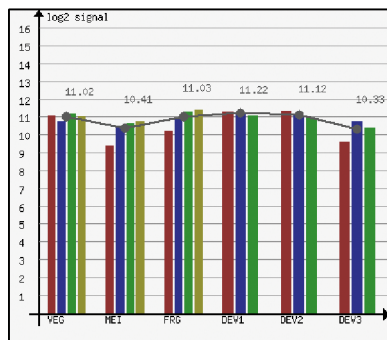

Supplement: Additional file 3 — Figure S2. Subfunctionalization of ohnologs of the recent WGD. Log-transformed autogamy time course for a family of 4 ohnologs, taken from ParameciumDB gene pages. The colored bars represent different biological replicates for each time point (see ParameciumDB gene pages for details). From top to bottom, the ParameciumDB accession numbers are GSPATG00035959001, GSPATG00008040001, GSPATG00007828001 and GSPATG00005693001. Only the top two genes are differentially expressed during autogamy, and are found in the "early peak" and "intermediate induction" clusters respectively. The dendrogram drawn on the left indicates the recent and intermediate WGD relationships of the 4 genes. [file 1471-2164-11-547-S3.PDF]
